# Flourishing older Canadians: What characteristics are associated with complete mental health?

**DOI:** 10.1371/journal.pone.0344898

**Published:** 2026-04-08

**Authors:** Daniyal Rahim, Shannon K. Halls, Ying Jiang, Esme Fuller-Thomson

**Affiliations:** 1 Department of Leadership, Higher, and Adult Education, Ontario Institute for Studies in Education, University of Toronto, Toronto, Ontario, Canada; 2 Factor-Inwentash Faculty of Social Work, University of Toronto, Toronto, Ontario Canada; 3 Applied Research Division, Center for Surveillance and Applied Research, Public Health Agency of Canada, Ottawa, Ontario, Canada; 4 Institute for Life Course and Aging, University of Toronto, Toronto, Ontario, Canada; University of Botswana, BOTSWANA

## Abstract

This study identified the factors associated with the absence of psychiatric disorder (APD) in the past year and complete mental health (CMH), among a nationally representative sample of Canadian adults aged 65 and older. APD is defined as the absence of suicidality, mental illness, and substance use disorder in the past year. CMH is defined as a combination of the following three characteristics: (1) APD; (2) life satisfaction or happiness almost every day in the past month and (3) social and psychological well-being almost every day in the past month. Data on 2,024 older respondents were obtained from the 2022 Mental Health and Access to Care Survey. The sample was analyzed using bivariate analyses and multivariate logistic regression models. Factors associated with ADP and CMH among older adults included being male, married, having social support, reporting religious or spiritual beliefs to be important, and having excellent self-reported health. Respondents without chronic pain, who had no difficulties in instrumental activities of daily living, had no sleep problems, and no history of depression, anxiety, or substance use disorder also had increased odds of both ADP and CMH. Findings from this study identify key factors associated with APD and CMH in older adults, which could inform the development of targeted public health interventions aimed at enhancing CMH within this population.

## Introduction

Over the past few decades, mental health research has primarily focused on mental illnesses, including depression, anxiety disorders, and schizophrenia [1]. However, emerging literature has increasingly emphasized the need to reconceptualize mental health not solely as the absence of psychopathology, but as the presence of positive psychological attributes [2]. Within the domain of positive psychology, Corey Keyes [1] introduced the construct of “flourishing” to describe a state of optimal mental health characterized by high levels of emotional, psychological, and social well-being. To operationalize this construct, Keyes [2] developed three distinct dimensions of well-being in the past month: 1) emotional well-being (e.g., happiness or life satisfaction); 2) psychological well-being (e.g., life is meaningful and has purpose); and 3) social well-being (e.g., the presence of supportive interpersonal relationships). A closely related concept to flourishing, called complete mental health (CMH), is defined as having flourishing mental health in the past month as well as the absence of psychiatric disorder (APD) such as anxiety, depression, bipolar disorder, suicidality and substance use disorder for the past year [1,3].

The shift toward examining concepts like flourishing and CMH has provided a more holistic approach to mental health research beyond merely examining the absence of psychopathology [1]. For instance, studies have shown that those who are flourishing have improved physical health, lower risk of mental illness, and reduced all-cause mortality risk [4].

Previous literature examining CMH using a nationally representative sample of Canadian adults has identified several factors consistently associated with the development of CMH across various sub-populations including adults with adverse childhood experiences (ACEs) [5–7], those with chronic physical health conditions such as stroke, cancer, COPD, and chronic pain [8–11], and those with a lifetime history of mental health problems including major depressive disorder, anxiety disorder, bipolar disorder, and suicidality [12–16]. These factors can be grouped into categories; 1) Socio-demographic characteristics associated with CMH include older age, being White, and higher education and income. 2) The prevalence of CMH is typically lower among those with a lifetime history of mental illness including depression, anxiety disorders and bipolar disorders and drug and alcohol use disorders. 3) With respect to health behaviors, physical activity has been associated with a higher prevalence of CMH, while smoking and obesity have been associated with a lower prevalence of CMH. 4) Both social support and the use of spirituality to deal with daily challenges are coping strategies associated with higher prevalence of CMH. 5) ACEs, including childhood exposure to domestic violence, sexual abuse, and/or physical abuse, are associated with a lower prevalence of CMH.

Although numerous studies have identified that older adults have an increased prevalence of CMH compared to younger adults [12,13,16,17], there is a lack of information on the particular characteristics associated with APD and CMH among older adults. Additionally, while APD is a component of CMH, examining factors associated with APD and CMH separately allows for a more comprehensive understanding of mental health [3]. Identifying distinct and overlapping predictors or APD and CMH may help identify which factors are associated with different aspects of mental health and allow for more informed treatment and health-promotion strategies. Therefore, the aim of the current study is to determine what factors are associated with 1) APD and 2) CMH, among a nationally representative sample of older Canadian adults.

## Methods

### Study population

This study was a secondary analysis of the 2022 Mental Health and Access to Care Survey (MHACS) [18], which used a representative sample of community-dwelling Canadians aged 15 and older living in the ten provinces, excluding people living on reserves and other Aboriginal settlements [19]. The subsample used for the current sample was restricted to MHACS respondents aged 65 and older. The survey response rate was 30.4% among those aged 65 and older [19]. If fewer than 50 respondents had missing data on a particular variable, those respondents with missing values were excluded from the analyses. For variables with 50 or more respondents with missing data, a separate “missing” category was created, and those respondents with missing data on that variable were included in the analysis. A consistent sample size was used for all analyses, to allow for direct comparison across different models. The final sample for this study consisted of 2,024 Canadians aged 65 and older. This study involved a secondary analysis of the MHACS publicly available data that had been fully anonymized, with all personally identifiable information removed prior to access. As such, ethical review and approval by the University of Toronto Research Ethics Board was not required. The original data collection, including the ethical oversight and informed consent procedures, was conducted by Statistics Canada—the national statistical agency of Canada, analogous to the United States Census Bureau. The original MHACS data was gathered by Statistics Canada between March 17, 2022, and July 31, 2022. EFT accessed the public use data for research purposes June 6, 2023. The public use data contains no information that would allow the identification of individual participants.

### Measures

#### Outcome Variables.

As has been discussed elsewhere [14] the construct of APD over the past 12 months was operationalized as the absence of suicidal ideation, bipolar disorder, major depressive episode, anxiety disorders, and substance use disorder, including alcohol, cannabis, and other illicit drugs. These diagnoses were derived using the World Health Organization Composite International Diagnostic Interview (WHO–CIDI) [19], a structured diagnostic instrument that produces DSM-IV and ICD-10 diagnoses. The WHO–CIDI has demonstrated robust validity and reliability, with concordance coefficients ranging from 0.73 for anxiety and phobic disorders to 0.78 for depressive disorders, and up to 0.83 for substance use disorder [20,21]. Test-retest and inter-rater reliability exceed 90% [20].

Complete Mental Health (CMH) was conceptualized as a binary construct consisting of three components: (1) APD, as defined above; (2) emotional well-being, including measures of happiness or life satisfaction; and (3) social and psychological well-being. The latter two components were assessed using the 14-item Mental Health Continuum – Short Form (MHC-SF) [22], a validated measure of positive mental health across emotional (e.g., “How often did you feel happy or satisfied with your life during the past month?”), social (e.g., “How often did you feel that you had something important to contribute to society?”), and psychological domains (e.g., “How often did you feel that you liked most parts of your personality?”) [22,23]. Participants were classified as being in CMH if they met the APD criteria and endorsed at least one of the three indicators of emotional well-being (i.e., happiness, or life satisfaction in the past month) and at least six of the 11 items reflecting social and/or psychological well-being as occurring “every day” or “almost every day” in the past month.

#### Covariates.

The following variables were included in the analyses: self-reported sex at birth (male versus female), self-reported race/ethnicity [White (includes Indigenous respondents) versus Visible Minority], immigrant status (immigrant versus Canadian-born), marital status (married/common-law versus widowed/divorced/never married), income (<$40,000, $40,000-$79,999, $80,000 or more), and highest level of education achieved (high school graduate or equivalent or less vs more educations). Physical activity (whether the respondent participated in any moderate or vigorous physical activity in the 7 days preceding the survey). Social support was based upon how much respondents agreed that they could “count on people that you know to help you deal with your biggest stress” (strongly agree/agree, neither agree nor disagree, disagree/strongly disagree, missing data). Spiritual coping was based upon the question “In general, how important are religious or spiritual beliefs in your daily life?” (very important, somewhat or not very important, not important at all). Chronic Pain was assessed through two questions: Respondents were determined to be regularly in pain if they responded negatively to the question “Are you usually free of pain or discomfort?”. Those who responded no were asked “How many activities does your pain or discomfort prevent?” The response categories of the combined variable were the following: no pain/no activities prevented by pain, pain prevents few/some/most activities, missing data. Self-reported health was based upon the question “In general, how is your health?” (excellent, very good, good, fair, poor). Limitations in instrumental activities of daily living (IADLs) were posed to respondents by asking “In the last 30 days, how much difficulty did you have in taking care of your household responsibilities?” [no difficulties versus yes (mild/moderate/severe difficulties)]. Sleep problems were assessed through responses to how often respondents had trouble going to sleep or staying asleep (none/a little/some of the time versus most of the time/all of the time). The present study also examined the extent of ACEs among respondents. ACEs were defined as how many of 6 types of childhood adversities were reported (0 versus 1 or more).

Geographic residence was assessed using population size groupings, categorized as rural areas (population <1,000; reference category), small population centres (1,000–29,999), medium population centres (30,000–99,999), and large urban population centres (100,000 or greater). Lifetime anxiety disorders and depressive disorders were assessed based on the WHO-CIDI [19] lifetime criteria for Generalized Anxiety Disorder and Major Depressive Episode. Two forms of substance use disorders were also included based on WHO-CIDI [19] “Lifetime Drug Use Disorder (including Cannabis)” and “Lifetime Alcohol Use Disorder”.

### Statistical analyses

Descriptive univariate and bivariate statistics, including frequencies/percentages and chi-square tests, were used to describe the study sample and the association between each of the variables and the outcomes of interest, APD or CMH. For each outcome of interest, two binary logistic regression models were conducted. Model 1 included the following variables (described above); sex, race/ethnicity, immigrant status, marital status, income, education level, physical activity, spiritual coping, social support, chronic pain, sleep problems, limitations in IADLs, self-reported health and ACEs. Model 2 included all variables from model 1, with the addition of geographical classification, lifetime history of major depressive disorder, generalized anxiety disorder, and drug or alcohol use disorder. Bootstrap weights provided by Statistics Canada were used to estimate 95% confidence intervals (CI) and account for the complex survey design. All analyses were completed using Stata version 17.

## Results

Table 1 presents the bivariate analyses between the predictor variables and the outcome variables of interest (APD and CMH). There were significant associations for both outcomes for gender, marital status, income level, physical activity, social support, spirituality, chronic pain, self-reported health, limitations in IADLs, sleep problems, ACEs, lifetime history of depression, anxiety, drug and alcohol use disorder. Education level was not significantly associated with either APD or CMH. Race/ethnicity was significantly associated with APD, but not CMH. Immigrant status was significantly associated with CMH (p = 0.05) but not APD (p = 0.3). The prevalences of APD and CMH were higher among respondents who were male and married. Regarding race, respondents who were visible minority members had a higher prevalence of APD compared to white respondents. For income, higher earnings were associated with better CMH, whereas the lowest income group showed the highest prevalence of APD. Respondents who had moderate physical activity within the last 7 days, those with social support, those who reported that religion and/or spirituality was important to them, and those who reported good to excellent health had a higher prevalence of both APD and CMH. Furthermore, APD and CMH prevalence rates were also higher among those without ACEs, residents of rural areas or medium population centers, respondents without chronic pain, and those with no limitations in IADLs, no sleep problems, and no lifetime history of major depressive disorder, generalized anxiety disorder, drug use disorder, or alcohol use disorder.

**Table 1:** Bivariate analyses examining the prevalence of Absence of Psychiatric Disorders in the Past Year (APD) and Complete Mental Health (CMH) (n = 2,024).

Variables	n	% with APD	P-values for APD	% in CMH	P-values for CMH
**Gender**			<0.001		<0.001
Male	1041	96.2		77.3	
Female	983	92.3		70.9	
**Race/Ethnicity**			0.002		0.8
Visible minority	630	95.7		74.2	
White	1394	93.8		73.9	
**Immigrant Status**			0.3		0.05
Canadian born	1213	94.0		74.4	
Immigrant	811	94.5		72.8	
**Marital Status**			<0.001		<0.001
Never married/Widowed/Separated/Divorced	658	92.0		67.8	
Married/Common-law	1366	95.3		77.3	
**Income Level**			0.005		<0.001
< $40,000	352	95.3		69.4	
$40,000 to $79,999	627	93.5		73.8	
$80,000 or higher	1045	94.0		75.9	
**Education Level**			0.3		0.9
High school diploma or equivalency certificate or less	677	93.8		74.0	
Trades certificate, College, CEGEP, or University certificate AND bachelor’s degree AND HIGHER	1347	94.3		73.9	
**Moderate Activity within the last 7 days**			<0.001		<0.001
Yes	1522	95.0		76.9	
No	502	91.6		65.6	
**Can count on people to cope with stress**			<0.001		<0.001
Strongly agree/Agree	1603	94.7		76.5	
Neither agree nor disagree	163	94.1		63.7	
Disagree/Strongly disagree	150	84.1		51.1	
Don’t know/refuse/not stated	108	99.3		80.3	
**Spirituality Level**			<0.001		<0.001
Very important	860	93.3		78.1	
Somewhat or not very important	868	95.3		72.9	
Not at all important	296	92.5		67.3	
**Chronic Pain**			<0.001		<0.001
No pain or no activity prevented by pain	1627	96.1		77.6	
Pain prevents few, some, or most activities	397	86.4		59.6	
**Self-perceived Health**			<0.001		<0.001
Excellent	325	99.2		88.3	
Very Good	712	96.2		77.1	
Good	708	92.0		70.1	
Fair	224	91.7		64.4	
Poor	55	78.8		43.9	
**Limitations in instrumental activities of daily living (IADLs)**			<0.001		<0.001
Yes	389	85.4		56.7	
No	1635	96.4		78.3	
**Sleep Problems**			<0.001		<0.001
none to some sleep problems	1731	96.0		76.6	
most or all of the time sleep problems	293	84.1		59.5	
**Adverse Childhood Experiences (ACEs)**			<0.001		<0.001
No ACEs	1011	95.8		77.7	
1 or more ACEs	926	92.2		70.0	
Missing	87	96.5		71.4	
**Geographical Classification**			<0.001		<0.001
Rural area (less than 1,000)	377	95.5		79.4	
Small population centre (1,000–29,999)	253	93.4		75.2	
Medium population centre (30,000–99,999)	161	95.8		79.4	
Large urban population centre (100,000 or greater)	1233	93.4		70.1	
**Major Depressive Disorder (MDD)**			<0.001		<0.001
No MDD	1917	96.3		75.6	
Major depressive disorder during lifetime	107	60.4		47.3	
**Generalized Anxiety Disorder**			<0.001		<0.001
No	1902	96.9		76.4	
Yes, in a lifetime	122	56.3		39.3	
**Drug Use Disorder**			<0.001		<0.001
No	1960	94.5		74.6	
Yes, in a lifetime	64	83.0		54.0	
**Alcohol Use Disorder**			<0.001		<0.001
No	1750	95.0		75.2	
Yes, in a lifetime	274	89.5		66.7	

Table 2 shows the logistic regression results examining factors associated with APD among older adults. In model 1, being male, married/common-law, having social support, reporting spirituality is very or somewhat important to them, having no chronic pain, having excellent, very good, or fair self-reported health, no IADL limitations, having no ACEs, and being free of sleep problems were all significantly associated with increased odds of APD in the past 12 months. Additionally, both higher income categories ($40,000-$79,999: OR=0.45, 95% CI: 0.35–0.57 and $80,000 + : OR=0.35, 95% CI: 0.27–0.46) were associated with significantly decreased odds of APD compared to those with income under $40,000. Model 2 added the following variables to the analysis: geographical classification, a lifetime history of major depressive disorder, generalized anxiety disorder, drug use disorder, and alcohol use disorder. Most significant associations from Model 1 remained significant, but many associations were substantially attenuated such as the odds ratios for marital status [OR from 1.80 to 1.30], social support for those who strongly agree/agree [OR from 1.97 to 1.44], no chronic pain [OR from 1.60 to 1.35], self-reported excellent health [OR from 9.00 to 6.15], very good health [OR from 2.32 to 1.62], and fair health [OR from 1.84 to 1.66] categories, and freedom from sleep problems [OR from 2.54 to 1.92]. However, the association for having no ACEs was no longer significant in Model 2. Additionally, those who did moderate activity within the last 7 days had significantly decreased odds of APD in model 2 [OR=0.69, 95% CI: 0.53–0.88], but there was no significant relationship in model 1. Those without a lifetime history of major depressive disorder or generalized anxiety disorder had significantly higher odds of APD (7.45, 95% CI: 5.78–9.60 and 11.91 95% CI: 9.47–14.97, respectively). Those with no history of drug use disorder also had significantly higher odds of APD [OR=1.78, 95% CI: 1.21–2.61], while history of alcohol use disorder was not significantly associated with APD.

**Table 2:** Logistic Regression Analyses for Absence of Psychiatric Disorders in the Past Year (APD) (n = 2024).

Variables	AOR (95% CI) Model 1	AOR (95% CI) Model 2
**Gender**		
Male	1.73 (1.47-2.04)***	1.89 (1.53-2.34)***
Female	1.00 (Ref)	1.00 (Ref)
**Race**		
Visible minority	1.35 (0.99-1.82)	1.33 (0.98-1.80)
White	1.00 (Ref)	1.00 (Ref)
**Immigrant Status**		
Canadian born	1.00 (Ref)	1.00 (Ref)
Immigrant	1.11 (0.86-1.41)	0.82 (0.63-1.07)
**Marital Status**		
Never married/Widowed/Separated/Divorced	1.00 (Ref)	1.00 (Ref)
Married/Common-law	1.80 (1.48-2.20)***	1.30 (1.03-1.64)*
**Income Level**		
Less than$40,000	1.00 (Ref)	1.00 (Ref)
$40,000 to $79,999	0.45 (0.35-0.57)***	0.55 (0.43-0.71)***
$80,000 or higher	0.35 (0.27-0.46) ***	0.50 (0.37-0.68)***
**Education Level**		
High school diploma or equivalency certificate or less	1.00 (Ref)	1.00 (Ref)
Trades certificate, College, CEGEP, or University certificate AND bachelor’s degree AND HIGHER	1.13 (0.95-1.35)	1.16 (0.95-1.41)
**Moderate Activity within the last 7 days**		
Yes	1.01 (0.83-1.22)	0.69 (0.53-0.88)**
No	1.00 (Ref)	1.00 (Ref)
**Can count on people to cope with stress**		
Strongly agree/Agree	1.97 (1.59-2.45) ***	1.44 (1.08-1.92)*
Neither agree nor disagree	2.43 (1.76-3.36)***	2.65 (1.84-3.82)***
Disagree/Strongly disagree	1.00 (Ref)	1.00 (Ref)
Don’t know/refuse/not stated	14.42 (7.34-28.29)***	11.35 (6.03-21.35)***
**Spirituality Level**		
Very important	1.29 (1.03-1.62)*	1.67 (1.27-2.20)***
Somewhat or not very important	2.12 (1.70-2.66)***	1.91 (1.47-2.47)***
Not at all important	1.00 (Ref)	1.00 (Ref)
**Chronic Pain**		
No pain or no activity prevented by pain	1.60 (1.34-1.92)***	1.35 (1.11-1.64)**
Pain prevents few, some, or most activities	1.00 (Ref)	1.00 (Ref)
**Self-perceived Health**		
Excellent	9.00 (5.50-14.74)***	6.15 (3.24-11.66)***
Very Good	2.32 (1.65-3.28)***	1.62 (1.04-2.52)*
Good	1.30 (0.96-1.77)	0.96 (0.63-1.46)
Fair	1.84 (1.28-2.65)**	1.66 (1.08- 2.56)*
Poor	1.00 (Ref)	1.00 (Ref)
**Limitations in instrumental activities of daily living (IADLs)**		
Yes	1.00 (Ref)	1.00 (Ref)
No	2.17 (1.77-2.65)***	2.86 (2.31-3.53)***
**Sleep Problems**		
none to some sleep problems	2.54 (2.14-3.03)***	1.92 (1.56-2.36)***
most or all of the time sleep problems	1.00 (Ref)	1.00 (Ref)
**Adverse Childhood Experiences (ACEs)**		
No ACEs	1.61 (1.35-1.92)***	1.07 (0.88-1.30)
1 or more ACEs	1.00 (Ref)	1.00 (Ref)
Missing	2.16 (1.45-3.21)***	0.92 (0.62-1.35)
**Geographical Classification**		
Rural area (less than 1,000)		1.00 (Ref)
Small population centre (1,000–29,999)		0.84 (0.63-1.11)
Medium population centre (30,000–99,999)		0.93 (0.67-1.28)
Large urban population centre (100,000 or greater)		0.92 (0.73-1.16)
**Major Depressive Disorder (MDD)**		
No MDD		7.45 (5.78-9.60)***
Major depressive disorder during lifetime		1.00 (Ref)
**Generalized Anxiety Disorder**		
No		11.91 (9.47-14.97)***
Yes, in a lifetime		1.00 (Ref)
**Drug Use Disorder**		
No		1.78 (1.21-2.61)**
Yes, in a lifetime		1.00 (Ref)
**Alcohol Use Disorder**		
No		1.17 (0.86-1.59)
Yes, in a lifetime		1.00 (Ref)

AOR, adjusted odds ratio.

**p < .01; ***p < .001.

Table 3 shows the logistic regression results for the association between CMH, and the same variables included in Table 2. Being male, being married/common-law, having social support, perceiving spirituality as very or somewhat important, reporting that their health as fair or better, experiencing no chronic pain, having at least a moderate level of physical activity, having no IADL limitations, being free from sleep problems, and no ACEs were all significantly associated with higher odds of CMH in both models. Unlike the findings for APD (Table 2), income was not significantly associated with CMH. In model 2, several associations remained significant but were somewhat attenuated compared to model 1, including marital status [OR from 1.49 to 1.33], social support (strongly agree/agree [OR from 2.35 to 2.14] and neither agree nor disagree [OR from 1.51 to 1.44]), no chronic pain [OR from 1.31 to 1.23], all categories of self-reported health (excellent [OR from 4.01 to 3.59], very good [OR from 2.09 to 1.90], good [OR from 1.70 to 1.55], and fair [OR from 1.76 to 1.64]), freedom from sleep problems [OR 1.45 to 1.30], and having no ACEs [OR from 1.35 to 1.22]. Spirituality associations remained strongly associated with CMH, with high importance of religion/spirituality [OR=2.07, 95% CI: 1.82–2.36] increasing slightly from model 1. Additionally, those without a lifetime history of major depressive disorder or generalized anxiety disorder had 1.77 (95% CI:1.45–2.15) and 3.01 (95% CI: 2.59–3.50) times the odds of CMH, respectively. Those without a lifetime history of drug use disorder [OR=1.35, 95% CI: 1.09–1.66] and alcohol use disorder [OR=1.17, 95% CI: 1.04–1.32] also had significantly higher odds of CMH. Finally, living in a large urban population center was associated with significantly lower odds of CMH compared to living in a rural area [OR=0.62, 95% CI: 0.56–0.69].

**Table 3:** Logistic Regression Analyses for Complete Mental Health (n = 2,024).

Variables	AOR (95% CI) Model 1	AOR (95% CI) Model 2
**Gender**		
Male	1.30 (1.19-1.41)***	1.30 (1.18-1.43)***
Female	1.00 (Ref)	1.00 (Ref)
**Race**		
Visible minority	0.92 (0.79-1.07)	1.03 (0.88-1.21)
White	1.00 (Ref)	1.00 (Ref)
**Immigrant Status**		
Canadian born	1.00 (Ref)	1.00 (Ref)
Immigrant	0.90 (0.79-1.02)	0.91 (0.80-1.03)
**Marital Status**		
Never married/Widowed/Separated/Divorced	1.00 (Ref)	1.00 (Ref)
Married/Common-law	1.49 (1.34-1.64)***	1.33(1.20-1.47)***
**Income Level**		
Less than $40,000	1.00 (Ref)	1.00 (Ref)
$40,000 to $79,999	0.99 (0.87-1.12)	1.04 (0.92-1.18)
$80,000 or higher	1.00 (0.88-1.14)	1.12 (0.98-1.28)
**Education Level**		
High school diploma or equivalency certificate or less	1.00 (Ref)	1.00 (Ref)
Trades certificate, College, CEGEP, or University certificate AND bachelor’s degree AND HIGHER	0.95 (0.87-1.04)	1.00 (0.91-1.09)
**Moderate Activity within the last 7 days**		
Yes	1.23 (1.11-1.35)***	1.24 (1.12-1.36)***
No	1.00 (Ref)	1.00 (Ref)
**Can count on people to cope with stress**		
Strongly agree/Agree	2.35 (2.04-2.69)***	2.14 (1.85-2.47)***
Neither agree nor disagree	1.51 (1.25-1.82)***	1.44 (1.18-1.75)***
Disagree/Strongly disagree	1.00 (Ref)	1.00 (Ref)
Don’t know/refuse/not stated	2.66 (2.13-3.32)***	2.32 (1.85-2.92)***
**Spirituality Level**		
Very important	2.03 (1.79- 2.30)***	2.07 (1.82-2.36)***
Somewhat or not very important	1.40 (1.26-1.57)***	1.38 (1.23-1.54)***
Not at all important	1.00 (Ref)	1.00 (Ref)
**Chronic Pain**		
No pain or no activity prevented by pain	1.31 (1.19-1.45)***	1.23 (1.11-1.36)***
Pain prevents few, some, or most activities	1.00 (Ref)	1.00 (Ref)
**Self-perceived Health**		
Excellent	4.01 (3.11-5.18)***	3.59 (2.76-4.68)***
Very Good	2.09 (1.65-2.65)***	1.90 (1.48-2.43)***
Good	1.70 (1.36-2.13)***	1.55 (1.22-1.96)***
Fair	1.76 (1.39-2.22)***	1.64 (1.29-2.09)***
Poor	1.00 (Ref)	1.00 (Ref)
**Limitations in instrumental activities of daily living (IADLs)**		
Yes	1.00 (Ref)	1.00 (Ref)
No	1.76 (1.58-1.96)***	1.74 (1.57-1.94)***
**Sleep Problems**		
none to some sleep problems	1.45 (1.31-1.61)***	1.30 (1.16-1.43)***
most or all of the time sleep problems	1.00 (Ref)	1.00 (Ref)
**Adverse Childhood Experiences (ACEs)**		
No ACEs	1.35 (1.24-1.47)***	1.22 (1.12-1.34)***
1 or more ACEs	1.00 (Ref)	1.00 (Ref)
Missing	1.04 (0.86-1.24)	0.88 (0.73-1.07)
**Geographical Classification**		
Rural area (less than 1,000)		1.00 (Ref)
Small population centre (1,000–29,999)		0.88 (0.76-1.03)
Medium population centre (30,000–99,999)		0.94 (0.79-1.12)
Large urban population centre (100,000 or greater)		0.62 (0.56-0.69)***
**Major Depressive Disorder (MDD)**		
No MDD		1.77 (1.45-2.15)***
Major depressive disorder during lifetime		1.00 (Ref)
**Generalized Anxiety Disorder**		
No		3.01 (2.59-3.50)***
Yes, in a lifetime		1.00 (Ref)
**Drug Use Disorder**		
No		1.35 (1.09-1.66)**
Yes, in a lifetime		1.00 (Ref)
**Alcohol Use Disorder**		
No		1.17 (1.04-1.32)*
Yes, in a lifetime		1.00 (Ref)

AOR, adjusted odds ratio.

**p < .01; ***p < .001.

## Discussion

This study examined factors associated with both APD and CMH among older Canadians to help inform programs and interventions to support this population. Key factors associated with higher odds of APD and CMH among older adults include marital status, social support, religion and spirituality, fair or better self-reported health, no limitations in IADLs, absence of chronic pain or sleep problems, no lifetime history of major depressive disorder and generalized anxiety disorder, and no lifetime history of drug use disorder.

Coping strategies such as accessing social support and use of religion/spirituality were important factors associated with APD and CMH. Individuals with social support had at least twice the odds of achieving APD and CMH, compared to those without, consistent with previous research [16]. For example, a study among those with chronic and debilitating pain found that social support was strongly associated with CMH [11]. Another study suggested pathways through which social support is associated with positive affect and reduced symptoms of mental illness such as anxiety and depression [24]. They suggest that social support improves mental health and positive affect, in part, through the impact that support has on perceived stress, in that social support changes how individuals perceive and handle stress, leading to stress reduction and generally improved mental health [24]. Additionally, it is believed that social support better fosters effective coping strategies, acting as a buffer against poor mental health in individuals coping with stressful situations [25].

Individuals who reported being married or in a common-law relationship had nearly 40% higher odds of CMH and 31% higher odds of APD in the fully adjusted model, consistent with previous studies which found marriage to be positively associated with achieving CMH [16,26]. Marital status may be a particularly salient form of social support.

Respondents who reported that religion and spirituality were very important had more than twice the odds of CMH, while those who reported that religion and spirituality were “somewhat important” had two times the odds of APD. Several previous studies have explored the importance of religious and spiritual practices for CMH [6,8,16]. A systematic review found that older adults who valued religious and spiritual practices had a lower likelihood of mental illness (i.e., anxiety and depression) and greater positive well-being, such as life satisfaction and meaning in life [27]. Religion and spirituality may promote APD and CMH by offering individuals guidance and coping resources during challenging periods through social support in religious communities [27]. It is also believed that religion and spirituality help people find meaning in times of suffering, which may reduce symptoms of depression and promote APD or CMH by altering the interpretation of adversity in ways that provide purpose and understanding of negative life experiences [27].

Absence of chronic and debilitating pain was found to be associated with a higher odds of both APD and CMH (16). This finding was in line with our previous research on adults 20 and older which found that absence of such pain was significantly associated with 34% higher odds of CMH even after taking into account lifetime mental health and substance use disorders [16]. There is a high comorbidity between chronic pain and mental health outcomes such as depression. Studies have shown a bi-directional relationship between these factors, such that mental illness can worsen pain perception, and chronic pain can also contribute to chronic stress and hopelessness leading to poor mental health [28].

In the current sample of older adults, the odds of APD and CMH were higher among those without IADL limitations. This finding aligns with previous research, including a study of individuals with childhood child welfare contact, which also reported a similar finding with respect to the IADL limitation and CMH [6]. Furthermore, another study of a nationally representative Canadian sample of adults aged 20 and older found that those without functional limitations had almost double the odds of CMH than those with functional limitations [16]. Pain has been identified as a potential mediator in the relationship between functional limitations and depression and/or anxiety [29], and it is common for those with functional limitations to experience pain, which is often associated with worsening mental health [28]. Additionally, loss of independence or a reduced sense of mastery (feeling of control over one’s life) associated with limitations in IADLs may contribute to declines in mental health [29,30].

Individuals who reported having sleep problems were less likely to experience APD in both models. These findings align with prior research demonstrating a negative association between insomnia and life satisfaction [31–33]. Longitudinal research has provided robust evidence that inadequate sleep significantly increases the risk of developing depression and anxiety disorders [34]. Given that older adults undergo various physiological changes that can adversely affect sleep [35], it is essential to further investigate effective interventions that can support healthy sleep patterns in this population.

Moderate physical activity was associated with increased odds of CMH in both model 1 and 2 but was unexpectedly associated with decreased odds of APD model 2. The finding that physical activity is associated with increased odds of CMH is consistent with previous literature [9,12]. It is believed that physical activity promotes CMH through various mechanisms, such as promoting resiliency and confidence [36], boosting the production of endorphins and other neurochemicals to improve mood and reduce stress [37], lowering the production of stress inducing chemicals like cortisol [38], and improving sleep quality [37]. Thus, it is unclear why moderate activity would be associated with *decreased* odds of APD. Future research is needed to shed light on this perplexing finding.

It is not surprising that those who had never had a previous diagnoses of major depressive disorder nor generalized anxiety disorder had much higher odds of APD and CMH in the year preceding the study. By definition, those with CMH must be free from mental illness in the past year, and there is substantial literature which suggests that previous mental illness is a strong predictor for future mental illness [39–41].

Residing in a large urban population center was significantly associated with a lower odds of CMH. This finding is in line with previous research which has discovered that urban living is often associated with an increased risk of psychological distress and mental health disorders including anxiety and depression [42–44]. Studies have shown that this increased risk may be in part due to factors such as social isolation, air pollution, and increased noise, which can contribute greatly to stress and decreased mental health [43,45,46]. Income was significantly associated with CMH. However, interestingly, it was found that higher income levels ($80,000 or higher) compared to lower income levels (less than $40,000) were associated with decreased odds of ADP. This is in contrast to what has been generally found in the literature, that lower income is associated with increased odds of mental illness [47]. It is unclear why this may be the case, and future research may be needed to further explore this relationship.

### Strengths and limitations

A key strength of this study is its use of a representative sample of older Canadians, enhancing the generalizability of the findings. However, it is also important to discuss the limitations of the study when considering our findings. First, the study was limited to exploring only factors that were available within the dataset. There are many additional factors including cognitive impairment, or personality characteristics such as high levels of conscientiousness and extraversion and low levels of neuroticism that are important contributors to mental health and flourishing that we were unable to examine in the current study [48]. Second, the survey data on lifetime substance misuse and mental illness, although using reliable WHO-CIDI questions, may be subject to recall bias, particularly for episodes that occurred many decades earlier. Such an error would probably bias the findings of the study towards the null. Thirdly, this study used cross-sectional data, which prohibits our ability to make causal inferences regarding the association with examined factors and CMH or APD. However, the findings were consistent with several previous studies which included younger adults [5–10,12–14,16,49]. Fourth, the generalizability of the study findings to the indigenous population in Canada may be limited as we were not able to identify the proportion of indigenous respondents, and the MHACS data does not survey indigenous individuals on reserve.

### Conclusion and study implications

Despite these limitations, this study provides new insights into important factors which are associated with APD and CMH among older adults. Some of these are modifiable risk factors which could be the target of public health intervention aimed at increasing CMH among older adults. Increasing access to social support programming, utilizing and providing access to spiritual practices, and taking the time and resources to adequately address both physical and mental health concerns such as chronic pain, sleep problems, anxiety, and depression, may be important targets that could potentially increase the likelihood that older adults will achieve APD and CMH. Cognitive Behavioral Therapy is also a promising intervention worthy of further investigation as it has been shown to be effective for late life depression and anxiety disorders [50,51], chronic insomnia [52], chronic pain [53] and loneliness among older adults [54].
